# Multimodal characterization of flow-induced thrombus initiation and growth in extracorporeal membrane oxygenation

**DOI:** 10.1038/s41598-026-40177-3

**Published:** 2026-02-18

**Authors:** Frida Nilsson, Benedikt Sochor, Sara Henriksson, Stephan V. Roth, Lars Mikael Broman, Lisa Prahl Wittberg

**Affiliations:** 1 FLOW, Department of Engineering Mechanics, KTH, Stockholm, Sweden; 2 Deutsches Elektronen-Synchrotron DESY, Notkestr. 85, 22607 Hamburg, Germany; 3 Advanced Light Source, Lawrence Berkeley National Laboratory, 6 Cyclotron Rd, Berkeley, CA 94720 USA; 4 Umeå Centre for Electron Microscopy, Department of Chemistry, Umeå University, Umeå, Sweden; 5 Science for Life Laboratory, Department of Chemistry, Umeå University, Umeå, Sweden; 6 Department of Fibre and Polymer Technology, KTH, Stockholm, Sweden; 7 ECMO Centre Karolinska, Intensive Care and Transport, Pediatric Medicine and Intensive Care, Astrid Lindgren Children’s Hospital, Karolinska University Hospital, Stockholm, Sweden; 8 Department of Physiology and Pharmacology, Karolinska Institutet, Stockholm, Sweden

**Keywords:** Biophysics, Cardiology, Computational biology and bioinformatics, Diseases, Engineering, Medical research

## Abstract

In cases of severe cardiopulmonary failure, extracorporeal membrane oxygenation (ECMO) may be temporarily used as a life-saving support for cardiac and/or lung function. Operating under non-physiological flow conditions, characterized by elevated shear rates and stagnant flow zones, there is an increased risk of inducing thrombosis, bleeding and hemolysis. Pinpointing the underlying mechanism triggering the onset of thrombus formation may aid development of device design, as well as management of anti-coagulation, benefiting patient outcome. Here we present a combined methodology enabling a multiscale understanding of thrombus development. Two thrombi collected from different ECMO circuits were analyzed by computational fluid dynamics (CFD), ultra small angle X-ray scattering (USAXS) and scanning electron microscopy (SEM). USAXS quantified the density and bulk alignment of fibrin, building the thrombus scaffold structure. SEM provided information on cellular morphology and surface fibrin structure, and CFD identified regions in the ECMO circuit with high thrombotic potential. Together, this combined approach was able to link local flow conditions and the structural growth of thrombi in ECMO circuits.

## Introduction

Extracorporeal membrane oxygenation (ECMO) is a life-saving treatment used for critically ill patients with severe cardiac or respiratory failure when conventional intensive care is insufficient^1^. By extracorporeal oxygenation and carbon dioxide removal in blood, ECMO provides vital support during organ recovery or bridge to transplant. In the EMCO circuit, blood continuously circulates between the patient and the extracorporeal circuit composed of several artificial components, including pump, membrane lung (ML), cannulas, tubing and connectors. The ECMO circuit exposes the blood to non-physiological flow conditions, such as elevated shear rates and stagnant flow^2–4^. These mechanical factors contribute to coagulation and platelet activation, increased inflammation and risk of hemolysis via mechanisms partly through both the intrinsic and extrinsic coagulation pathways^3,5,6.^

The classic Virchow’s triad describes the fine balance between the risk factors for thromboembolism, with stagnant flow, endothelial injury, and hypercoagulability^7,8^. However, it does not account for the high shear situations that develop in ECMO circuits. High shear rates may activate platelets and thus initiate thrombus formation. This highlights the need to expand traditional thrombosis models to include mechanical triggers intrinsic to extracorporeal circulation^9^. To mitigate the clotting risk during ECMO, a systematic continuous anticoagulation treatment is necessary, most commonly heparin. However, anticoagulants introduce other risks, such as bleeding and heparin-induced thrombocytopenia, making it challenging to balance thrombotic and hemorrhagic risks^10,11^.

The von Willebrand factor (vWf) protein exhibits a shear sensing behavior in native flow. Recently, the importance of vWf and its response to situations of high shear and turbulent flow was reported^12–14^. In high shear flows ($\dot{\gamma }>5000$ s$^{-1}$), vWf will form net-like structures capturing platelets that may initiate rapid thrombus growth even at low platelet counts^9,15^. Further increasing the shear rate, Liu et al. (2022) found vWf to unfold in milliseconds at 10,000 s$^{-1}$, displaying the short time scale in which the process can be initiated^16^. Zhang et al. (2024) found that a rapid change in shear activated the GPIb$\alpha$ platelet receptor, and made it even more susceptible to bind to vWf^17^. Current knowledge also acknowledges that stresses marginally larger than physiological levels are harmful to red blood cells (RBCs), platelets and vWf^18,19^. From this perspective, the flow fields developing in the ECMO circuit components are particularly challenging as the flow-induced stresses acting on the blood range from physiological up to 100,000 s$^{-1}$^3,20^.

Typically, thrombi formed under high shear are referred to as “arterial” thrombi and characterized by high platelet and fibrin content, whereas “venous” thrombi, regarded to be formed under low-shear conditions, are composed by mainly RBC and fibrin. The most common approach to investigate thrombi is by electron microscopy, assessing surface morphology or histology^21–23^. Recent works have applied computed tomography (CT) for volume characterization of human thrombi after thrombectomy (microCT) and in-vitro thrombus growth in ML (cone beam CT)^24,25^. Another approach is to study individual blood components, to understand how the surrounding flow impacts the local growth process of the thrombus. It is known that fibrin, the last clotting factor in the coagulation cascade, creates the scaffolding of the thrombus. Fibrin has a full length of 45 nm with an axial repeating unit of 22.5 nm. This knowledge has been used to study pure fibrin gels with ultra small angle X-ray scattering (USAXS). Jansen et al. (2020) studied randomly arranged fibrin gels and found isotropic concentric fibrin rings, where the axial order Bragg peaks were more pronounced for denser fibers and the peak width indicating crystallite size depended on the protein density^26^. Vos et al. (2020) considered fibrin alignment at different strains and found the networks to progressively align along the principal direction of strain^27^. Gersh et al. (2010) reported that fibrin fibers to orient in the flow direction in the location where they were formed. Moreover, the alignment increased with increasing flow rates, with a maximum wall shear rate of 20 s$^{-1}$ in their study^28^. Thus, understanding the flow field can provide important feedback for understanding thrombus growth in the ECMO circuit, highlighting the importance of thrombus growth connected to shear dynamics in the circuit components.

Today there is no methodology providing the information needed to understand details of thrombus composition and morphology combined with sources of initiation and development. Here we suggest a combined approach using USAXS for fibrin orientation and density, scanning electron microscopy (SEM) for surface morphology and component composition and computational fluid dynamics (CFD) for evaluation of flow structures and stress dynamics. To illustrate methodological feasibility and potential, two thrombi were assessed: one collected from the inlet of a CentriMag pump (Abbott Laboratories, Pleasanton, CA, USA) and the other collected from the tubing between the outlet of a CentriMag pump and the inlet of the ML. The results show that the combination of USAXS, SEM and CFD enable a multiscale understanding of thrombus initiation and growth, linking fibrin structure alignment to local flow dynamics. This approach provides mechanical insights into thrombus growth under shear conditions and highlights critical flow regions in the ECMO pump.

## Results

**Fig. 1:**
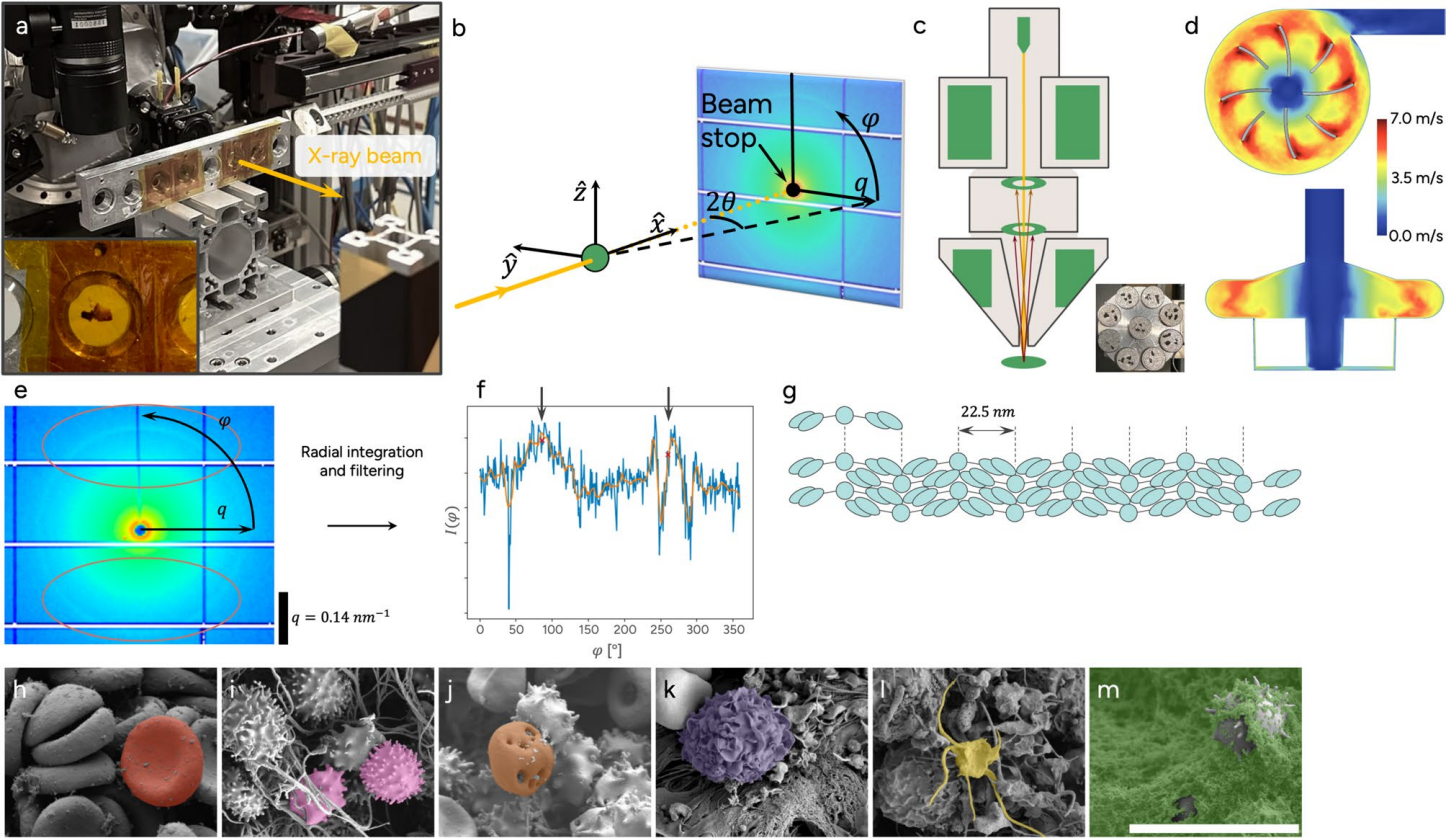
Experimental setup, data reduction and studied blood components. (**a**) Thrombus collected from the inlet of a centrifugal pump mounted in a sample holder pending analysis with ultra small angle X-ray scattering (USAXS). (**b**) Typical USAXS 2D pattern with coordinate system and beam stop, captured with a PILATUS 2M detector (DECTRIS, Baden-Dättwil, Switzerland). The sample is marked in green. (**c**) Scanning electron microscope (SEM) schematic with sample holder. (**d**) Phase averaged velocity field magnitude of the centrifugal blood pump (CentriMag pump (Abbott Laboratories, Pleasanton, CA, USA). (**e**) USAXS 2D detector image with typical ring pattern from fibrin alignment. The red ellipses indicate areas with increased intensity, $I_{fib}$, along $\varphi$ on the fibrin ring. (**f**) Intensity as a function of $\varphi$ after performing radial integration and filtering (orange) to extract fibrin alignment, with peaks indicated with black arrows. (**g**) Fibrin schematic showing the internal repeating unit of 22.5 nm. Scanning electron microscopy (SEM) images for (**h**) normal red blood cell (red), (**i**) two different morphologies of Echinocytes (pink) in fibrin threads, (**j**) hemolytic red blood cell (brown), (**k**) leukocyte (purple), (**l**) activated platelet (yellow) and (**m**) fibrin sponge, platelet fragments (green). The scale bars correspond to 10 $\mu m$ and is valid for panels (**h**)–(**m**).

The two thrombi were collected from two different patients. The first, Thrombus A, was found in the pump inlet and the second, Thrombus B, was collected from the tubing between the pump outlet and the ML inlet. USAXS was used to obtain information on bulk fibrin alignment, and SEM was used to study the surface and cell morphology. These results were interpreted in conjunction with results from CFD studies on the flow structures in the region where the respective thrombus was collected. An overview of the combined approach and the blood components observed (RBCs, echinpocytes, hemolysis, leukocyte, activated platelets and fibrin sponge/platelet aggregates), is provided in Fig. 1, and described in the “Methods” section.

### Thrombus A: inlet recirculation

**Fig. 2:**
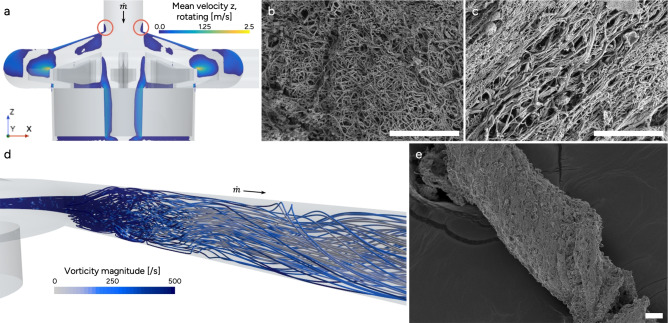
Fibrin alignment depends on local flow conditions. (**a**) Phase averaged axial velocity field for 3000 rpm and 1 L/min, indicating flow pushing up through the inlet causing a recirculation zone (pink circles). (**b**, **c**) Thrombus A: Unstructured (isotropic) and highly aligned fibrin, at different positions on the surface, from the thrombus collected in the pump inlet, (scale bar: 10 $\mu m$). (**d**) Swirling flow in pump outlet flow, for 3000 rpm and 1 L/min. (**e**) Thrombus B collected from the tubing between the pump outlet and membrane lung inlet, shows a twisted thrombus structure resembling the flow structures in panel (**d**) (Scale bar: 20 $\mu m$).

**Fig. 3:**
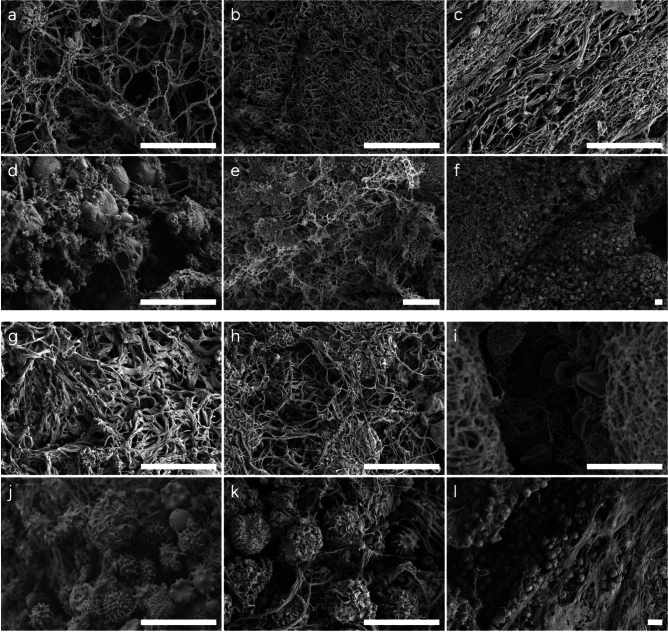
Thrombus surface morphology. (**a**–**f**) **Thrombus A**, thrombus from the pump inlet. (**a**), (**b**) highly varying fibrin density with no particular direction of alignment. (**c**) Highly aligned fibrin. (**d**) Spherocytes captured in fibrin mesh covered in platelet fragments. (**e**)-(**f**) Echinocytes captured in pore structures in the fibrin scaffolding. (**g**-**l**) **Thrombus B**, thrombus from tubing between the pump outlet and the membrane lung inlet. (**g**), (**h**) fibrin threads of different thickness with several captured leukocytes in (**h**). (**i**) Regular red blood cells, stomatocytes and echinocytes captured in crevasses in the fibrin structure. (**j**)–(**l**) Leukocytes captured in the fibrin mesh, with (**l**) being partly covered in platelet fragments or fibrin sponge. (scale bar: 10 $\mu m$).

**Fig. 4:**
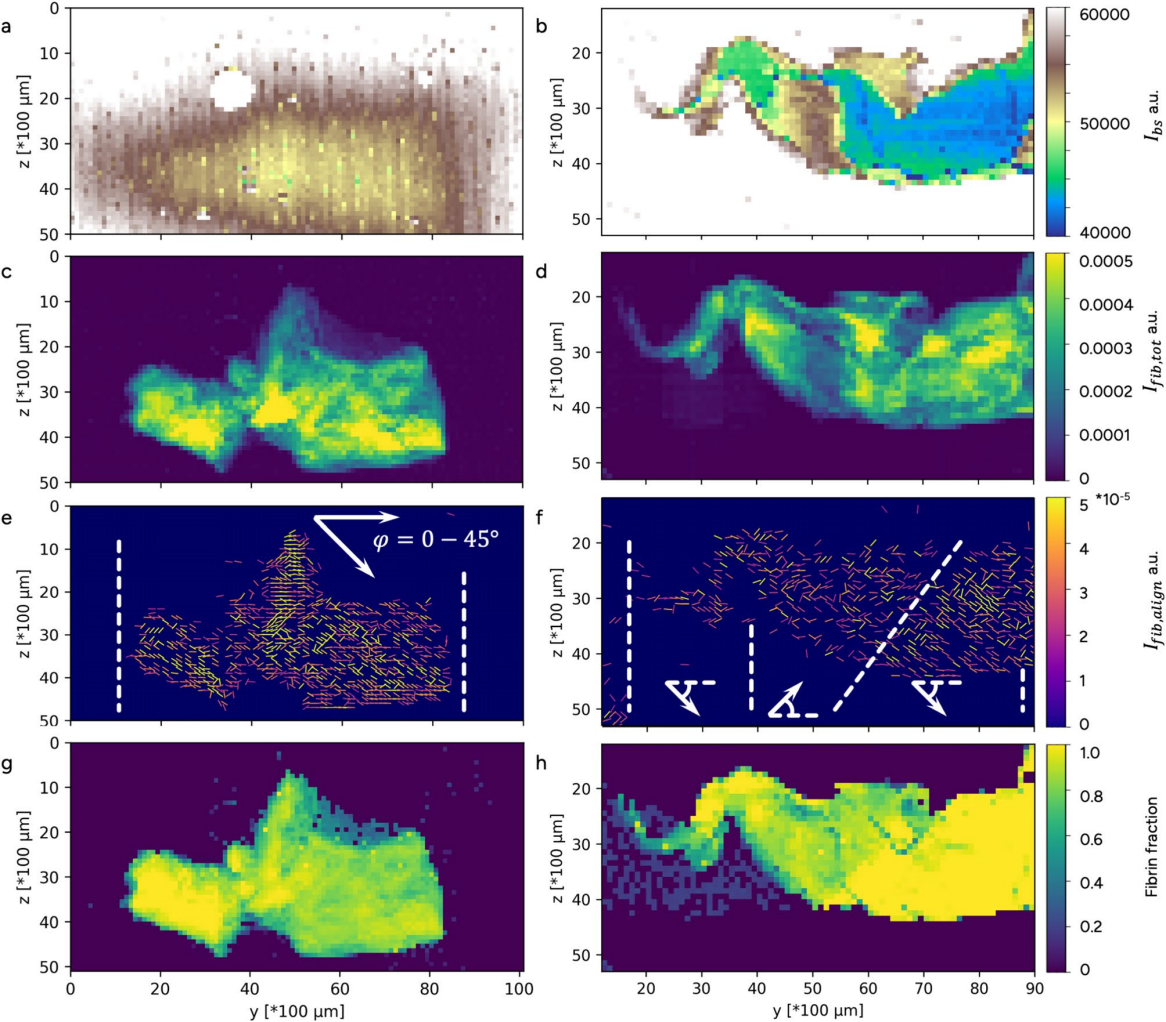
Thrombus fibrin structure. (**a**, **b**) Beam stop diode intensity for (**a**) Thrombus A and (**b**) Thrombus B, with the white circle at $y=40$ and $z=20$ is an air bubble. (**c**, **d**) Fibrin intensity for (**c**) Thrombus A and (**d**) Thrombus B. e&f) Fibrin alignment with its alignment height for (**e**) Thrombus A and (**f**) Thrombus B. The arrows give a qualitative indication of the overall alignment in different sections of the thrombi. In (**e**) and (**f**) the color indicates peak intensity and the orientation of the line indicates the orientation of the fibrin. (**g**, **h**) Fraction of fibrin scattering to total recorded scattering for (**g**) Thrombus A and (**h**) Thrombus B.

Thrombus A was collected at the pump inlet, from the circuit used on Patient A. Through CFD, a recirculation zone was identified in this area of the pump, Fig. 2a. Generally, surface characterization with SEM displayed a random fibrin structure of varying density with some regions of more aligned fibrin, Fig. 3a–c. Moreover, the fibrin mesh showed a mix of both clean areas, and areas covered with platelet fragments, Fig. 3b and d respectively. The USAXS data showed a general direction of horizontal alignment, revealing that fibrin preferred to align in one direction through the thrombus, Fig. 4e. The thrombus gave a strong fibrin signature and an overall fibrin content of minimum 70 %, Fig. 4c and g. Regarding cell morphology, Thrombus A also contained echinocytes and some spherocytes mixed with regular RBCs, Fig. 3d–f.

### Thrombus B: swirling flow at pump outlet

Thrombus B was collected from the tubing between the pump outlet and the inlet of the ML, from the circuit used on Patient B. The RBC morphology varied, with regular, echinocytes, stomatocytes and some signs of hemolysis being present, Fig. 3i–l. Thrombus B contained leukocytes, visible as spherical particles with a ruffled surface, clustered on the surface and bound in the fibrin mesh, see Fig. 3h, j–l. Both RBCs and leukocytes were captured in pockets along the structure of the thrombus. The fibrin pattern on the outer surface showed no preferred alignment direction, Fig. 3g, h. However, some regions exhibited a twisted arrangement that became apparent when viewed on a larger scale, Fig. 2e. This twisted pattern corresponded well to the swirling flow detected in the pump outlet using CFD, Fig. 2d. The analysis of fibrin alignment with USAXS showed a shifting behavior, varying from negative $45^\circ$ to positive $45^\circ$, with the dominant direction of alignment being less pronounced in the middle of the thrombus from USAXS, around $y=3700$ $\mu$m to $y=6000-7500$ $\mu$m Fig. 4f. This shifting behavior correlated with the surface structure, Fig. 2e. Overall, the USAXS analysis of Thrombus B showed a dominant fibrin content (Fig. 4h) but with a weaker fibrin signature (Fig. 4d) indicating a less dense fibrin network. The alignment was less strong compared to Thrombus A, Fig. 4e, f.

**Fig. 5:**
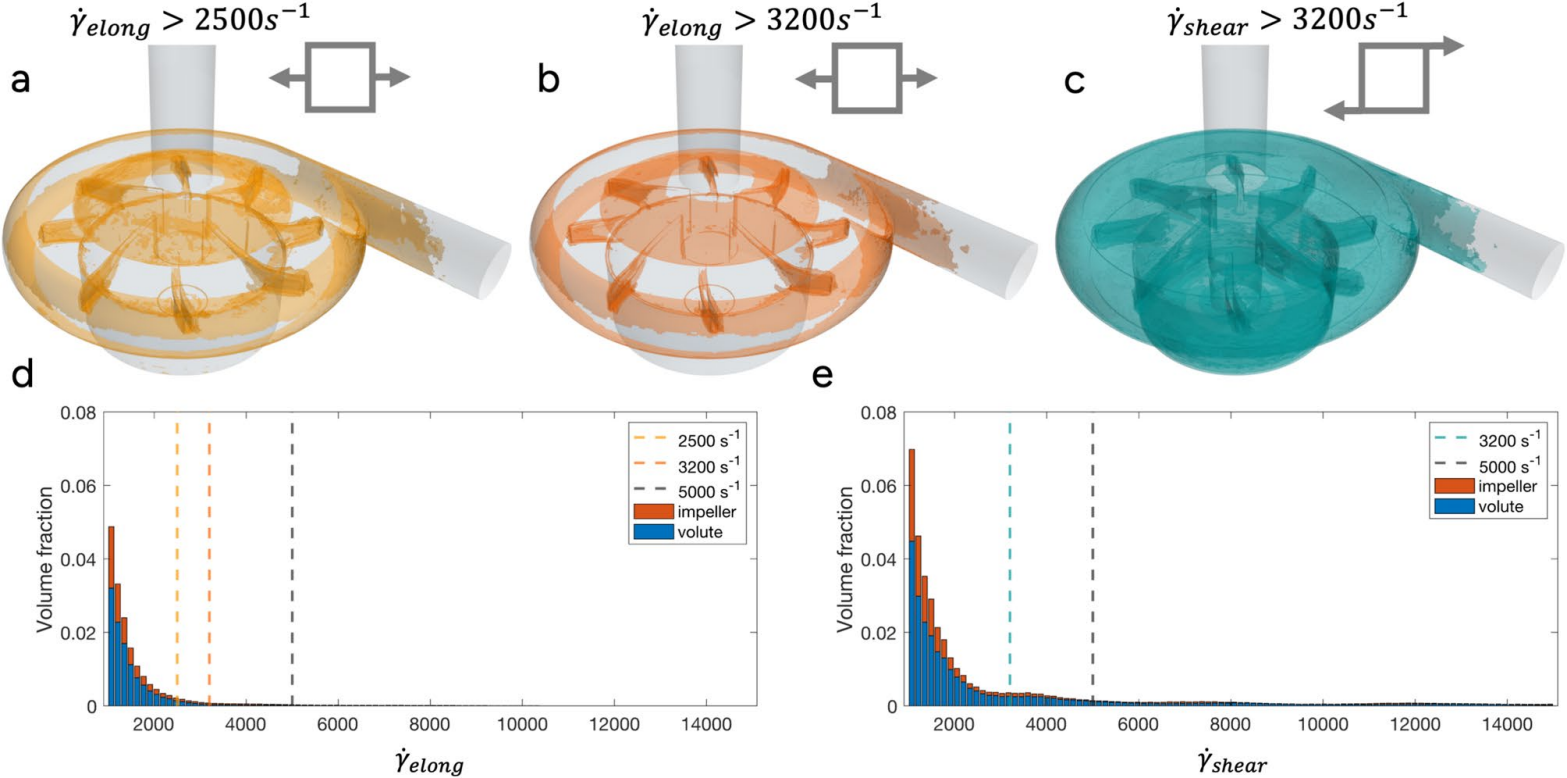
Shear rates in flow. (**a**–**c**) Phase averaged elongational and off-diagonal shear stresses for the CentriMag pump. (**d**, **e**) Elongational and off-diagonal volume fractions to the entire pump volume.

By splitting the elongational ($\dot{\gamma }_{elong}$) and off diagonal ($\dot{\gamma }_{shear}$) components of the shear rate tensor in a cylindrical coordinate system (CFD), estimations of zones more likely to exhibit elongational flow were detected, Fig. 5 and Eqs. (3, 4). Elongational shear, at values which may cause vWf unfolding (i.e. $\dot{\gamma }_{elong}>2000 s^{-1}$), was found in 3% of the pump volume, and 8.2% of the pump volume experienced off diagonal shear stresses above the recommended 12 Pa^19^.

## Discussion

Human thrombi from ECMO circuits used for life support were collected, the thrombus structure investigated and the results evaluated in the context of the flow conditions under which the thrombus had been formed. The morphology of individual blood cells found in the samples was also analyzed.

Thrombus A, collected from the pump inlet, a region where previous studies have shown a recirculation zone with low momentum flow pushing up along the top of the pump casing and along the sides of the pump inlet, particularly at low flow rates^29^. The mechanisms for this kind of backflow were further described by Nilsson et al.^20^. In this case, the pump flow rate was low, 1 L/min at 3000 rpm. CFD results confirmed the suspected backflow at the pump inlet, Fig. 2a. Backflow is considered problematic from a thrombogenic perspective since this flow structure offers a zone where particles may reside for a longer time, as well as a strong shear layer at the boundary between the recirculation region and the incoming blood flow. Blood entering the pump inlet experiences an accelerated flow moving from laminar to transitional, where streamlines are tightly organized and velocity gradients are strong and relatively uniform. Thrombus A had a dense fibrin network, probably resulting from the complex blood flow surrounding it, requiring the thrombus to be stiffer to resist disintegration or detachment from its base.

Studying human fibrin clots formed in a Chandler loop, Eyisoylu et al. (2024) observed that thrombi formed in flow were stiffer, more dense and had thinner fibrin fibers compared to no-flow conditions^30^. It was also reported that fibers formed under flowing conditions displayed more bundling, i.e. thin fibers bundling together and aligning in the same direction to form thicker fibers. For thrombi formed under stationary conditions, the structure was porous with thicker individual fibers. Zeng et al. (2023) studied blood clots from pig whole blood in Chandler loops and observed that increased shear rates resulted in a decrease in RBCs and an increase in fibrin^31^. Zeng et al. (2023) also reported that the RBCs were more tightly packed and that the leukocyte content was unaffected by shear. These observations may support the hypothesis that the aligned fibrin on the surface originates from fibrin growth within the shear layer formed between the accelerated inlet and the adjacent recirculation region, Fig. 3c. In contrast, less aligned fibrin with larger pores may have developed in the recirculation zone, a region observed to exhibit a larger turbulent kinetic energy and structures prolonging the residence time^2,20^.

Thrombus B, collected from the tubing between the pump outlet and the ML inlet, exhibited a partially twisted pattern according to the SEM images, a structural feature confirmed from the bulk study using USAXS analysis of fibrin. CFD data revealed swirling flow emerging from the pump outlet, suggesting that Thrombus B developed and increased in size while subjected to rotational motion. Given the thrombus formed downstream of the pump, the shear environment within the pump itself becomes critical to understand the thrombus initiation for Thrombus B. Time-averaged wall shear stress-based parameters are widely used to identify regions associated with elevated risk of blood damage or thrombus initiation^32,33^. However, in highly complex and non-physiological flows, as considered here, recent studies have shown these metrics to display large magnitude variability^34^. Inherent to the design these metrics also struggle to capture key mechanisms that occur in the bulk flow. In particular, they neglect the potential of particle damage or deformation away from the wall. As a result, important flow dynamics relevant to blood damage and thrombus formation may be filtered out.

The CFD results showed that the flow exceeded $\dot{\gamma }_{elong} > 2000s^{-1}$ in up to 3% of the pump volume, a limit established by Yeo et al. (2024)^18^ when examining the lower threshold for vWf unfolding in elongational flow. Notably, these high-strain zones were located in both the wake of the impeller blade tips and at the pump outlet, making these regions have a higher likelihood of vWf elongation and subsequent platelet activation.

Furthermore, the SEM examination showed abnormal RBCs in the shape of echinocytes and spherocytes. Pan et al. (2022) observed a 4.5-fold increase in echinocytes and a 2.3-fold increase in spherocytes from mechanical fatigue after 7 days of ECMO. This may explain the high prevalence of echinocytes and spherocytes in this study^35^. SEM also revealed leukocytes distributed throughout the thrombus, indicating an inflammatory component in the thrombus formation process. Neutrophils are known to be part of clot formation to support inflammation and contribute with metalloproteases and activation of a number of coagulation factors^36^. Moreover, monocytes contribute with tissue factor in the process of clot formation. Neutrophil extracellular traps (NETs) are formed during the quick process when an activated neutrophil releases its sticky DNA as a last resort to save the host when the neutrophil senses it will surpass^37^. NETosis has been shown both in the lung and other organs where these sticky NETs capture and kill bacteria extracellularly^38,39^. NETs have also been associated with clot formation^36^, and decreased NETosis may reduce thrombus formation^40^.

Squeezing the thrombi into envelopes for USAXS measurements could slightly alter their shape, but Vos et al. found no observable structural changes in their fiber or network structure until they exceeded a strain of 25%, which is much more than what is expected in this study^27^.

A significant portion of widely used models for both platelet activation and hemolysis are based on empirical data collected in Taylor-Couette-like systems^19^. These systems offer repeatable environments for studying shear induced blood damage. However, the simplified geometry introduces fundamental limitations when applying such models to complex clinical systems, such as ECMO circuits. The shear field in Taylor-Couette systems is mostly uniform and dominated by controlled off-diagonal shear, which lacks the multidirectional and elongational flow components present in ECMO circuits. Thus, the exposure history of blood components in the circuit, such as abrupt acceleration, recirculation zones and highly turbulent environments, is not reflected in these idealized systems. Froese et al. (2024) and Ding et al. (2015) reported that both pulsatile and steady flows in different Taylor-Couette-like system resulted in similar results for hemolysis^41,42^. However, these studies were performed on healthy donor blood with controlled hematocrit at room temperature, which may impact the clinical relevance in ECMO. For example, Schöps et al. (2021) found a large discrepancy between in-vitro and in-silico measurements of hemolysis in an ECMO circuit^6^. For low flow measurements, hemolysis was expected to be 3.5 times higher, whereas in high flow measurements expected to be 8.9 times higher. Both cases showed the same trend though, with hemolysis being higher for low flow conditions. This further highlights the importance of adapting current models to more complex systems, which in turn requires a deeper understanding of how individual blood components respond under varying stress conditions. The combination of USAXS and SEM provides the structural information that when coupled with the complex flow field from the realistic geometry, detailed in Fig. 1d, enables this coupling.

Both of our samples showed fibrin sponge or platelet fragments, Fig. 1m. Similar structures, “fibrin biofilm” have been observed in air-liquid interfaces in animal models, rationalized to protect the patient from microbial invasion^43^. However, there is also an another approach to biofilm, which may arise as a problem in healthcare, as in ECMO. Artificial surface, e.g. central venous access or ECMO cannula, may get colonized by, for example, staphylococci. When the colony has increased to a certain number, the colony forms a Quorum where the staphylococci start to communicate as an organism itself^44^. The quorum produces a protective cap impermeable to most known antibiotics. This cap is cytotoxic, and when the activated neutrophil attacks the cap, this will be its last mission ending with NETosis. The sticky DNA further augments the protective cap in benefit of the Quorum. Infected central lines are known for risk of clot formation. Exactly how the development of this type of biofilm impacts clot formation is an almost non-investigated field but appears to involve activation of specific coagulation factors and metalloproteases^45^. Platelet fragments or fibrin sponge were observed in both samples in this study. However, further studies are needed for increased understanding of details and interactions of different “biofilms”, focusing on if and how clot formation is triggered and evolving via these pathways.

In this work we show that combining SEM, USAXS and CFD can offer new insight into thrombus initiation, formation, and growth, especially by linking fibrin growth to the flow field dynamics. USAXS showed that the thrombus fibrin mesh can have an overall direction of alignment even though the surface is unordered. Moreover, USAXS also provided information on fibrin mesh construction. Combined with CFD, the fibrin mesh structure agreed well with the local flow field characteristics. USAXS also provided a measure for fibrin density, showing that the thrombus from the pump inlet was denser. The approach described here has the ability to identify both regions that promote thrombus growth and sources of activation in the flow field surrounding the thrombi. This in turn provides knowledge regarding the parts of the ECMO circuit components that are to be considered for re-design, and the details of the fluid structures to be avoided.

Future work will focus on further including and assessing patient variability, applying this method to a larger patient cohort. The goal is to form a thrombus risk assessment model, as well as a model for thrombus growth in extracorporeal life support devices.

## Methods

### Clinical material and sample preparation

The thrombi were collected from two pediatric patients after the circuit was removed at the end of treatment, which lasted for 6.5 to 8 days. The material presented in this study includes data from two male patients, patient A and patient B, according to Table 1.

The study was registered in the Database for Clinical Studies, Karolinska University Hospital, Stockholm, Sweden (K 2021-8409). Ethical approvals were granted by the Swedish Ethical Review Authority (EPM, Dnr: 2019-06446; 2020-04707). Informed consent was provided by the patients’ legal representatives for the collection of thrombi from the used ECMO circuit(s). Sample collection and preparation were conducted in accordance with institutional guidelines and applicable regulations.

**Table 1:** Patients with their respective circuit operating conditions.

Patient	Gender	Age	ECMO mode	Pump	ML	Flow rate [L/min]	rpm	Clot location
A	M	6y	VA	CentriMag	Eurosets	1.17-1.67	2800-3300	Pump inlet
B	M	3y	VV	CentriMag	HiLite 2400	1.35-1.62	2500-3000	Tubing between pump outlet and ML inlet

Abbreviations: M, male; y, years; VV, veno-venous; VA, veno-arterial; ML, membrane lung; rpm, revolutions per minute.

#### Sample preparation

After removing the ECMO circuit from the patient, it was rinsed from residual blood with lukewarm tap water. Thrombi were then carefully extracted. Each thrombus was immersed in sodium chloride solution (9 mg/mL) and cut into $\le 1\times 1$ mm strands and fixated ($2.5\%$ Glutaraldehyde, 0.1*M* Phosphate buffer, pH 7.4 ± 0.02). They were then stored in a refrigerator (+4 $^\circ$C) for 24 hours, after which the fixative was exchanged for a 0.1M Phosphate buffer solution (pH 7.40 ± 0.02). The samples were kept refrigerated pending analysis for a maximum of three months. Before analysis, each thrombus was split into two parts, one for analysis with SEM, and one for USAXS.

Thrombi studied with SEM were critically point dried (CPD) using a Leica CPD300 (Leica Microsystems, Wetzlar, Germany) and sputter coated with 15 nm of platinum (Quorum Technologies, Laughton, UK). No additional sample preparation was needed for the USAXS samples.

### Combined analysis for sample characterization and link to flow field

#### Small angle X-ray scattering

X-ray scattering was performed for ultra small angles at the P03 beamline at DESY in Hamburg, Germany. USAXS was chosen to capture larger structures, with a sample to detector distance (SDD) of 10 m and a beam energy of 11.9 keV corresponding to a wavelength of $\lambda =1.04$ Å. The beam size was $27\times 23$ $\mu m^2$. Calibrations to determine the SDD was done using rat tail collagen. Images of the scattered X-rays were captured on a hybrid photon counting X-ray detector, PILATUS 2M detector (DECTRIS, Baden-Dättwil, Switzerland, with pixel size $172\times 172$ $\mu m^2$). The scattering vector was calculated according to $q=(4\pi /\lambda )\sin \theta$, with $2\theta$ being the scattering angle. The images were captured with an exposure time of 1 s. The resulting q-range was $0.02-0.81 nm^{-1}$.

Each thrombus was put in a Kapton envelope (13 $\upmu m$ thick) taped to a sample rig and scanned in $100 \upmu m$ steps. These scans resulted in a set of approximately 5000 images each with a total acquisition time of approximately 10 h.

Data reduction was based on the Python library PyFAI^46^. By first masking the beam stop and detector grid and then selecting a thin segment around the fibrin ring ($q=0.28$ $nm^{-1}$), corresponding to the repeating unit of 22.5 nm, and performing azimuthal integration to the entire ring segment, a scatter profile was obtained. The scatter profile was normalized with its local beam stop intensity and the background was subtracted according to:

1 $$\begin{aligned} \begin{aligned} I_{fib} = \frac{I_{raw}}{I_{bs}}-\frac{I_{bg}}{I_{bs,bg}}, \end{aligned} \end{aligned}$$

where $I_{raw}$ is the profile obtained from azimuthal integration, $I_{bs}$ is the intensity recorded on the beam stop diode, $I_{bg}$ is a profile obtained from the liquid surrounding the thrombus and $I_{bs,bg}$ is the intensity recorded on the beam stop diode for the same location as $I_{bg}$. Savitzky-Golay filtering was applied to smooth the data but still preserve the peak shapes before peak extraction^47^. Peak positions were identified using the find_peaks function from the SciPy signal processing library, which detects local maxima by comparing each data point with its immediate neighbors^48^. From this maximum, $\phi (I_{fib,max})$,the fibrin alignment could be obtained as $\phi (I_{fib,align})=\phi (I_{fib,max}+90^\circ )$, Fig. 1e, f and fibrin schematic in Fig. 1g. This approach was used because of the low signal-to-noise ratio and the inherent unpredictable direction of alignment. Previous studies of fibrin gels have used methods based on the fact that the main direction of alignment is known, as the sample has been stretched in a specific direction^27^. The fibrin scatter fraction was evaluated as fibrin scattering compared to total scattering captured by the detector.

#### Scanning electron microscopy

The exterior and interior surfaces of the thrombus were examined by high-resolution SEM. For each sample, a set of images was collected to represent the overall surface structure of each thrombus, depending on homogenicity, to gain insight into cell morphology on both the surface and in crevices or tears. All images were collected with a field emission scanning electron microscope (FESEM) Merlin manufactured by Zeiss, see schematic in Fig. 1c. With a landing energy 3-4 keV and beam current of 100 pA. Samples were imaged using secondary electron detectors of in-lens or Everhart-Thornley type.

Figure 1h–m shows the different blood components observed in the two samples: regular RBCs, two types of echinocytes, hemolysis, leukocytes, platelets and fibrin sponge/platelet fragments.

#### Computational fluid dynamics

To model fluid dynamics in the CentriMag blood pump, commercial software StarCCM+ (Version 2402) was used. The flow was initiated with Reynolds-averaged Navier-Stokes with the k-$\omega$ closure model and thereafter resolved using Large eddy simulations (LES) with the WALE SGS model. The fluid was defined as Newtonian blood analog with a viscosity of 3.763 mPas and a density of 1060 kg/m$^3$. Further details and mesh validation are found in Berg et al. (2019)^49^. To estimate the elongational and off-diagonal shear rates in the pump flow the symmetric shear rate matrix was split:

2 $$\begin{aligned} \begin{aligned} \dot{\gamma } = \begin{bmatrix} \sigma _{rr} & \tau _{r \theta } & \tau _{rz}\\ \tau _{r \theta } & \sigma _{\theta \theta } & \tau _{\theta z}\\ \tau _{rz} & \tau _{\theta z} & \sigma _{zz} \end{bmatrix}. \end{aligned} \end{aligned}$$

providing an expression for the elongation:

3 $$\begin{aligned} \begin{aligned} \dot{\gamma }_{elong} = \sqrt{\sigma _{rr}^2 + \sigma _{\theta \theta }^2 + \sigma _{zz}^2}, \end{aligned} \end{aligned}$$

and an estimation of the shearing:

4 $$\begin{aligned} \begin{aligned} \dot{\gamma }_{shear} = \sqrt{2(\tau _{r \theta }^2 + \tau _{rz}^2 + \tau _{\theta z}^2)}. \end{aligned} \end{aligned}$$

## Data Availability

The datasets used and/or analyzed during the current study are available from the corresponding author on reasonable request.
